# Mapping cortical hubs in tinnitus

**DOI:** 10.1186/1741-7007-7-80

**Published:** 2009-11-23

**Authors:** Winfried Schlee, Nadia Mueller, Thomas Hartmann, Julian Keil, Isabel Lorenz, Nathan Weisz

**Affiliations:** 1 Department of Psychology, University of Konstanz, 78457 Konstanz, Germany

## Abstract

**Background:**

Subjective tinnitus is the perception of a sound in the absence of any physical source. It has been shown that tinnitus is associated with hyperactivity of the auditory cortices. Accompanying this hyperactivity, changes in non-auditory brain structures have also been reported. However, there have been no studies on the long-range information flow between these regions.

**Results:**

Using Magnetoencephalography, we investigated the long-range cortical networks of chronic tinnitus sufferers (n = 23) and healthy controls (n = 24) in the resting state. A beamforming technique was applied to reconstruct the brain activity at source level and the directed functional coupling between all voxels was analyzed by means of Partial Directed Coherence. Within a cortical network, hubs are brain structures that either influence a great number of other brain regions or that are influenced by a great number of other brain regions. By mapping the cortical hubs in tinnitus and controls we report fundamental group differences in the global networks, mainly in the gamma frequency range. The prefrontal cortex, the orbitofrontal cortex and the parieto-occipital region were core structures in this network. The information flow from the global network to the temporal cortex correlated positively with the strength of tinnitus distress.

**Conclusion:**

With the present study we suggest that the hyperactivity of the temporal cortices in tinnitus is integrated in a global network of long-range cortical connectivity. Top-down influence from the global network on the temporal areas relates to the subjective strength of the tinnitus distress.

## Background

Subjective tinnitus is defined as an auditory perception in the absence of any physically identifiable source for it. Almost everyone will experience some form of auditory phantom perceptions such as tinnitus at least once in their lifetime; in most of the cases this sensation vanishes within seconds or minutes. However, in 5 - 10% of the population in western societies the tinnitus persists for more than six months and usually remains chronic [1]. Those patients hear a constant ringing, buzzing or hissing in the ear and this perception is especially dominant when the patient is resting in a quiet environment. About 1 - 3% of the general population experience tinnitus as bothersome and complain that it affects their quality of life. Problems can include difficulties concentrating at work, a decrease in their social life, depression, insomnia or anxiety [2].

Tinnitus is typically associated with substantial damage to the hearing system such as a noise trauma or chronic noise exposure. This damage leads to plastic changes at various levels of the central auditory system and consequently enhanced neuronal synchrony and spontanous firing rate within the central auditory system. These changes have been well documented in animal and human studies and can be caused by different pathologies [3-7]. However, the mere hyperactivity of the central auditory system does not explain the diversity of tinnitus symptoms and the variability of the subjective tinnitus distress between patients. Thus, existing theories have stressed the importance of higher order association brain areas that could be involved in the processing of the tinnitus [4,7,8]. Cortical areas such as the frontal and the parietal lobe have been suggested to take part in a long-range neuronal network that is involved in the integration of sensory and emotional aspects of the tinnitus [4,7,8]. Furthermore it has been hypothesized that top-down mechanisms of this higher order network could modulate the activity of the auditory cortex [8]. This is in keeping with the model of the global neuronal workspace as suggested by Deheane and colleagues [9,10]. This global neuronal workspace is distributed over distant areas of the cortex, mainly in the parietal lobe, the frontal, and the cingulate cortex. According to this framework, conscious perception requires neuronal activity of the sensory areas together with an entry into this workspace realized by long-range cortical coupling. Top-down influence from the global workspace on the sensory cortices amplifies the neuronal activity within the respective sensory area. Using magnetoencephalographic recordings in the resting state we aimed to explicitly test these assumptions: 1) Is there neuromagnetic evidence for alterations of long-range cortical networks in tinnitus during the resting state? What brain areas and frequency bands are involved in this network ? 2) Is there evidence for a top-down influence of this global network on the auditory cortex and does it relate to the subjective degree of tinnitus distress ?

Abnormal patterns of long-range cortical coupling have been found in other pathologies and significantly contributed to their understanding. For instance, Le van Quyen et al. [11] found for the pre-ictal phase in epilepsy a decrease of long-range synchrony with the epileptic focus and this isolation was accompanied by an increase of local synchrony within the epileptic focus. Uhlhaas and colleagues [12] investigated schizophrenic patients during a Gestalt perception task and discovered a reduction of beta-band phase synchrony that might be related to their impairment in grouping stimulus elements together to form a coherent percept. Silberstein et al. [13] reported an increase of cortico-cortical coupling in Parkinson's disease that correlated with the strength of Parkinsonism. Therapeutic interventions such as the application of L-dopa or electrical stimulation of the subthalamic nucleus resulted in a reduction of the cortico-cortical coupling and Parkinson symptoms.

Resting-state recordings, collected when the participant is instructed 'to do nothing', are characterized by widely distributed networks of coherent brain activations [14-17]. Disturbances of this *default *network have been detected in disorders such as Alzheimer's or Parkinson's disease [13,18]. Since chronic tinnitus sufferers report an ongoing perception of the tinnitus sound that is most prominent when the environment is quiet, we expected to find abnormalities in the long-range couplings under resting conditions.

To investigate these abnormalities in magnetoencephalographic recordings we used a beamforming technique to reconstruct the brain activity in the source space and investigated the strength of coupling between them. Partial directed coherence (PDC) is a new approach to measure the effective coupling between multivariate time series. It is based on the concept of Granger causality and captures the direction of the information flow in the frequency domain [19,20]. Several groups have applied this concept successfully to investigate directed coherence between cortical regions: Supp et al. reported differences in the directed information flow during an object recognition task of familiar and unfamiliar objects using Electroencephalography (EEG) [21]; Babiloni et al. investigated directed cortical coherence patterns during commercial spots and emotional spots [22], and Gross et al. also used source reconstruction combined with PDC to analyze directed interareal communication using Magnetoencephalography (MEG) [23]. In the present study we used PDC to analyze the directed coupling between all pairs of voxels in a frequency range from 2 to 100 Hz.

**Figure 1:**
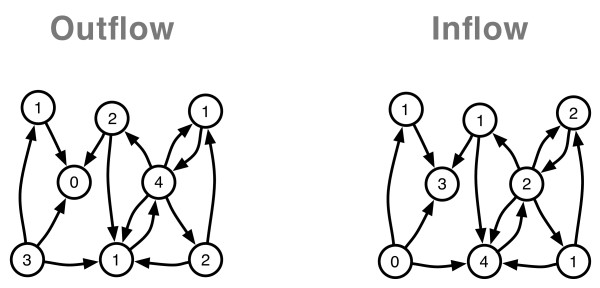
**Illustration of a directed network and the hubs within this network**. Left: The nodes are labeled with the hubdegree of the outflow (counting the arrow tails), Right: The nodes are labeled with the hubdegree of the inflow (counting the arrow heads).

Networks in general are comprised by two elements: nodes (here: voxels) and the links (here: coherence) between them. The importance of a node within this network varies with the number of connections it entertains with other nodes: i.e. a node with a large number of links receives information from many other nodes and/or influences many other nodes. These core structures within a network are called *hubs *and can be operationalized simply by counting the number of links (this is called the *degree *of the hub/node). In directed networks, the information on the directionality of the information flow is retained. The *inflow *to a voxel indicates that the activity of this voxel is driven by another voxel. Accordingly, a hub with a strong *outflow *describes that this voxel influences the activity of many other voxels (Figure 1). With this information we can identify the hubs within the network that are characterized by a strong *outflow *and/or by a strong *inflow*.

In this study, we modeled the resting-state networks in tinnitus and controls by pinpointing the core structures of inflow and outflow. First, we compared the inflow and outflow between the tinnitus and the control group and found differences in the long-range cortical networks under rest. Second, we correlated the strength of the inflow and outflow with the subjective strength of the tinnitus distress. We found that the inflow in the left and the right temporal cortex correlated positively with tinnitus distress. We interpret this result as reflecting the top-down influence on the auditory cortex that modulates tinnitus distress.

## Results

### Group differences

**Figure 2:**
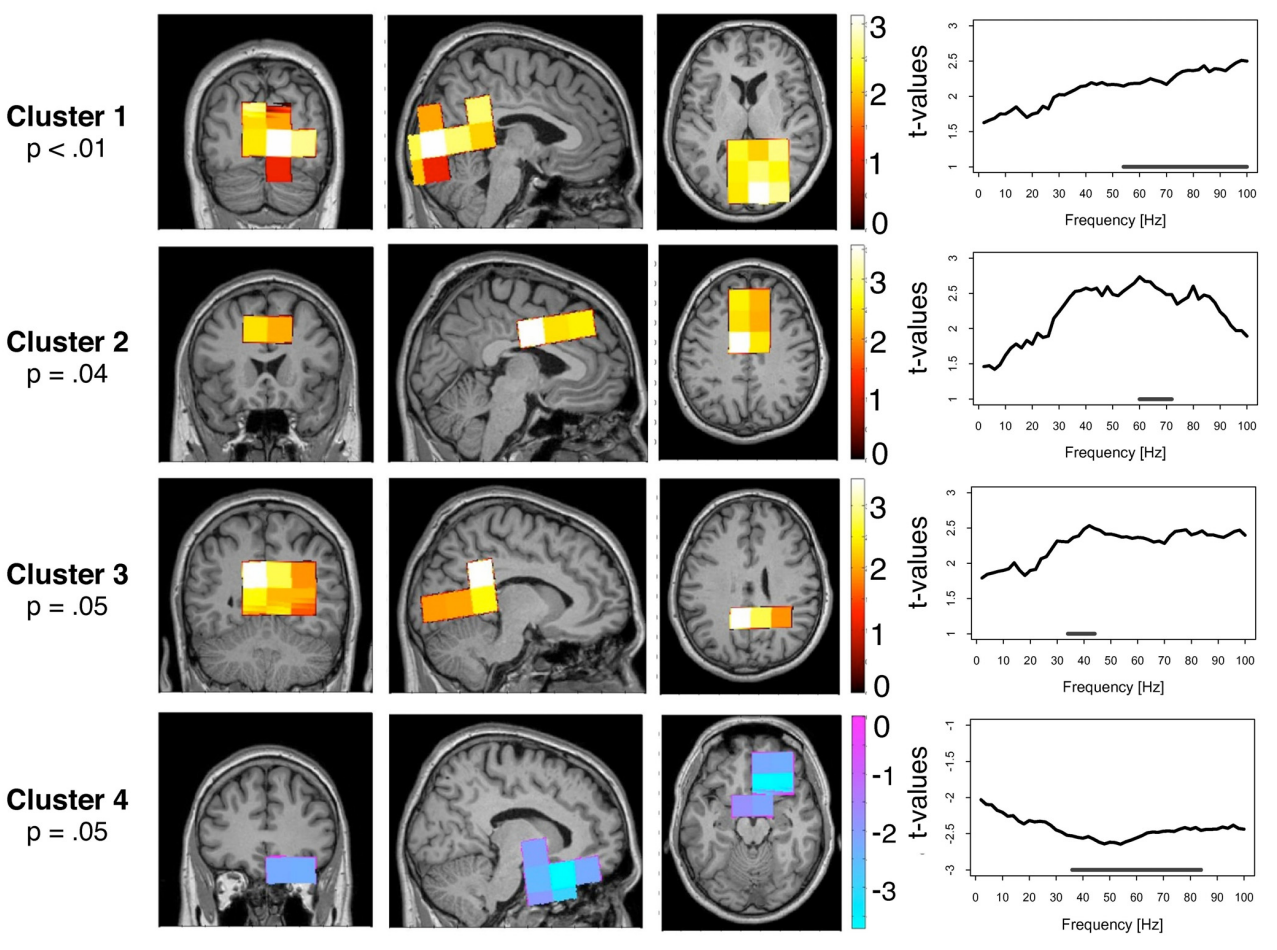
**Group difference for the outflow**. The strength of outflow describes how much the activity within the respective voxel drives the activity of other brain regions. Four clusters were found with a significant group difference between tinnitus and control participants. In the upper three clusters, the outflow was greater for the tinnitus group. In the lower cluster, the outflow of the tinnitus group was reduced. The location of the clusters are shown in the coronal, sagittal and horizontal view. The right column displays the significant frequency range of the clusters.

**Figure 3:**
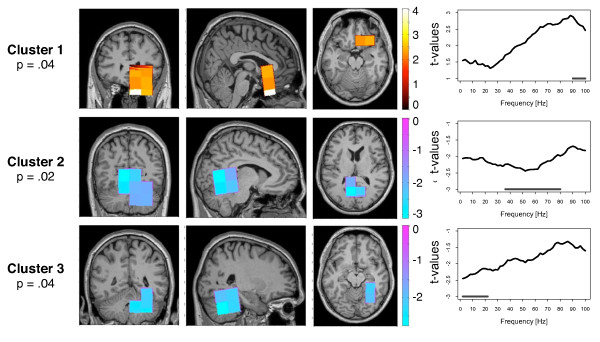
**Group difference for the inflow**. The strength of inflow describes how much the activity within the respective voxel is driven by the activity of other brain regions. Three clusters were found with a significant group difference between tinnitus and control participants. In the upper cluster, the inflow was greater for the tinnitus group. In the lower two clusters, the inflow of the tinnitus group was reduced. The location of the clusters are shown in the coronal, sagittal and horizontal view. The right column displays the significant frequency range of the clusters.

Primarily we were interested in alterations of long-range cortical networks in tinnitus. The sensor data were projected into source space using the linearly constrained minimum variance (lcmv) - beamformer technique onto a grid of 326 voxels with the size of 2 × 2 × 2 cm. Partial directed coherence was calculated in the frequency range of 2 - 100 Hz to estimate the directed coupling between all voxels. As an indicator for the long-range cortical networks we analyzed the core structures of inflow and outflow within these networks and mapped them on a standard brain. The inflow and the outflow were analyzed separately to investigate the main structures that are driving within this network as well as structures that are driven within the network. We calculated a nonparametric randomization test that controls for multiple comparison in order to identify spatial-spectral clusters of differences between the tinnitus and the control group. Figures 2 and 3 provide more detailed information on the significant clusters.

#### Outflow

In an analysis of the outflow of the cortical networks we found four significant clusters that differentiated between the tinnitus and the control group (see Figure 2). In the clusters 1, 2, and 3 the outflow is significantly increased for the tinnitus group. Cluster 1 was significant with a *P *- value <.01. The voxels of this cluster overlay a large area of the posterior part of the brain, including the cuneus, the posterior cingulum, and the precuneus. The cluster was slightly shifted to the right hemisphere. The outflow in this cluster was significantly increased in the higher frequency range from 54 - 100 Hz for the tinnitus group. The second cluster was significant with *P *= 0.04 and was localized in the prefrontal cortex. Outflows in the tinnitus group were stronger for the 30 - 90 Hz gamma band, but only the frequency range from 60 - 72 Hz survived the multiple comparison correction. The third cluster was marginally significant with *P *= 0.05. It was again located in the posterior part of the brain, approximately at the same location as the first cluster. The group difference was significant for the 34 - 44 Hz frequency range. Cluster 4 was the only cluster with negative t-values, i.e. the degree of the outflow was stronger for the control group. Cluster 4 was found in the orbitofrontal cortex (OFC) of the right hemisphere and was significant in the gamma frequency range from 36 - 84 Hz. The cluster differentiated significantly between the groups with a p-value of *P *= 0.05.

#### Inflow

For the inflows we found three clusters with a significant group difference (Figure 3). There was one positive cluster of inflows, with a *P*-value of *P *= 0.04. It was found in the orbitofrontal cortex. In the higher gamma frequency range tinnitus participants showed higher hubdegrees than control participants. Only the frequency range of 90 - 100 Hz the tinnitus group survived the multiple comparison correction. Cluster 2 and 3 were both clusters of negative t-values and were found in the posterior part of the brain around the posterior cingulum and also extending into the cerebellum. Inflows were significantly weaker for the tinnitus participants in those voxels. Cluster 2 was significant with *P *= 0.02 in the gamma frequency range from 36 to 80 Hz. Cluster 3 was significant with a *P*-value of *P *= 0.04 for lower frequencies (2 - 22 Hz).

### Correlation with tinnitus distress

**Figure 4:**
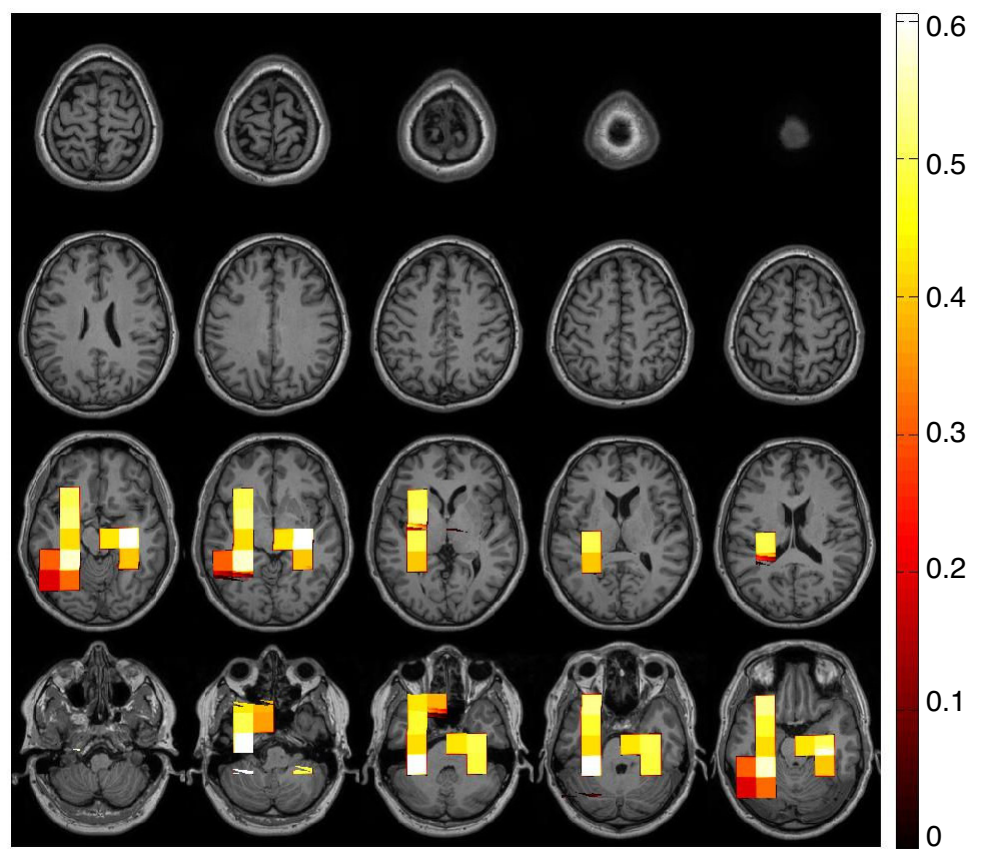
**Correlation of the strength of inflow with the subjective rating of the tinnitus distress**. The inflow to voxels in the left and the right temporal cortex correlated positively with the subjective strength of the tinnitus distress. No significant correlations between the outflow and the distress were found.

**Figure 5:**
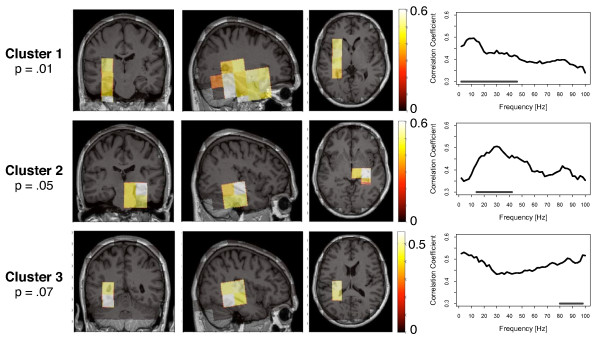
**Location and frequency band of the inflow-clusters that correlated with the individual tinnitus distress**. The stronger the inflow to the clusters, the stronger the subjective strength of tinnitus distress as assessed with a standard German Questionnaire. The location of the clusters are shown in the coronal, sagittal and horizontal view. The right column displays the significant frequency range of the clusters.

In this step of the analysis our goal was to investigate those parts of the cortical network that were modulated by the strength of the subjective distress of the tinnitus subjects. The tinnitus distress was assessed using the German version of the Tinnitus Questionnaire (Hogrefe, Göttingen, Germany, 1998) [24]. Using this instrument and the physiological measurements, we correlated the subjective tinnitus distress rating with the hubdegrees of the inflow and outflow for each frequency bin. As in the analysis on the group difference we used a cluster-based statistical analysis with correction for multiple comparisons. For the outflow we did not find any cluster that correlated significantly with tinnitus distress. For the inflows we found three clusters that correlated positively with the tinnitus distress rating. No clusters with negative correlations were found. Figure 4 gives an overview over all significant clusters and Figure 5 provides a more detailed view of the three clusters.

#### Inflow

We found three clusters of inflows that correlated positively with the subjective rating of the tinnitus distress. Stronger degrees of the inflows were associated with greater tinnitus distress. Cluster 1 was significant with *P *= 0.01 covering large parts of the left temporal cortex and also entering the frontal cortex to a small extend. The correlations were significant for the slow-wave frequencies, alpha, beta, and the lower gamma frequencies (2 - 46 Hz). The second cluster was located in the right temporal cortex and was significant with *P *= 0.05. In the frequency range of 14 - 42 Hz inflows correlated significantly with tinnitus distress. Cluster 3 was at the border of statistical significance (*P *= 0.07). This cluster was again located in the left temporal cortex and it covered the higher gamma frequencies from 80 - 98 Hz.

#### Origin of the inflow to the temporal clusters

**Figure 6:**
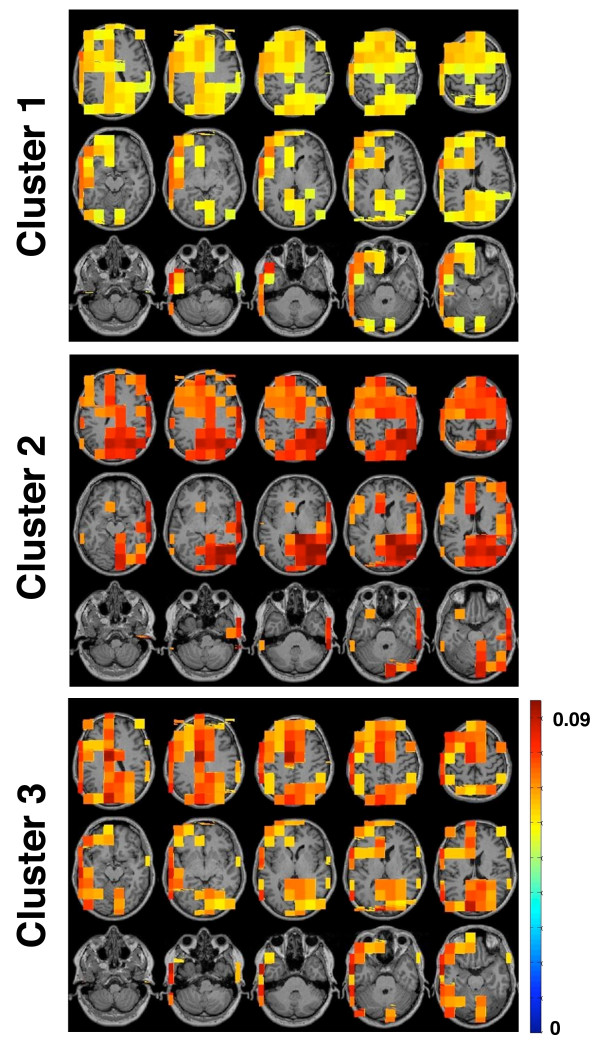
**Regions with Top-Down Influence on the Temporal Clusters**. The inflow to the clusters shown in figure 5 correlated with tinnitus distress. Here mapped the regions from where the top-down influence originated. Voxels with a low and putatively irrelevant influence on the clusters were masked.

Three clusters showed meaningful correlations of the strength of inflow with the subjective rating of the tinnitus distress. Thus, activity within these clusters was driven by other regions of the brain. In this final step of our analysis we were interested in their origin. Therefore, the raw PDC-values of all voxels with directed coupling to the respective cluster voxels were averaged across the given frequency range of this cluster. Figure 6 displays the mean influence of each voxel on the cluster voxels of Cluster 1, 2 and 3. Voxels with a low and putatively irrelevant influence on the clusters were masked for this figure. To do this we performed a bootstrapping with 1,000 resamples on this data to estimate the mean and the confidence interval. For the resampling we used the same logic as described in the data analysis section (Step 3, Point 1). Voxels that revealed mean PDC-values stronger than the higher limit of the confidence interval were plotted for this figure. The pattern of voxels influencing the temporal regions was similar for all three clusters. Firstly, they all received input from a large area in the frontal cortex. However, there is no influence from the right orbitofrontal cortex (Cluster 4 of the outflow; see Figure 2). Secondly, they all received influence from posterior voxels, approximately at the location of the outgoing clusters 1 and 3. Thirdly, they all received input from their directed neighborhood: The left temporal clusters (Cluster 1 and 3) received input from the adjacent left fronto-temporal region. Respectively, the right temporal clusters were influenced by the neighboring right fronto-temporal region.

## Discussion

In this study we found alterations in the functional coupling of long-range cortical networks between tinnitus and healthy control participants. Within this resting brain network we found regions with altered outflow and regions with altered inflow characteristics. A strong outflow in this context indicates that this brain area considerably influences the activity of other brain structures. In the tinnitus group two brain regions were identified with stronger outflow and one site with a weaker outflow. Stronger outflows were located in the prefrontal cortex and in the posterior part (parieto-occipital/occipital) of the brain. The weaker outflow was found in the orbitofrontal cortex. All of these changes in the outflow behavior were found for the gamma frequency band above 30 Hz. A strong inflow means that this brain area is strongly driven by other brain regions. With respect to the inflow characteristics we found two sites with significant group differences. The orbitofrontal cortex was receiving more inflow in the high frequency gamma range in the tinnitus group compared to the control group. Posterior parts of the cortex were receiving less inflow from other brain areas in a broad frequency range that included delta, theta, alpha, low beta and gamma frequencies. Furthermore, we found that the inflow to the temporal cortices correlates positively with the subjective ratings of the tinnitus distress. The more the activity in the temporal cortices was driven by other brain regions the stronger the subjective distress reported by the tinnitus subjects. Additionally, we also demonstrate that the inflow to the temporal cortex mainly originates from the prefrontal cortex and the posterior part of the brain; both are structures that we have characterized with a strong outflow within this network.

Thus we show significant alterations of the resting-state network in tinnitus. Although this was suggested earlier, empirical evidence was lacking to date. The prefrontal cortex, the orbitofrontal cortex, and the parieto-occipital region are important components within this network. The importance of the prefrontal cortex has previously been hypothesized by Jastreboff [4], who suggested that the prefrontal cortex integrates sensory and emotional aspects of tinnitus. In the present study we found that the prefrontal cortex in tinnitus strongly influences other brain regions of the network. In the 1960s it was shown that a disconnection of the prefrontal cortex results in a reduction of the annoyance of the tinnitus in most of the surviving patients [25]. Measuring the regional cerebral blood flow (rCBF) with Positron Emission Tomography (PET), Mirz and colleagues revealed a reduction of rCBF in the prefrontal cortex when the tinnitus was suppressed by lidocaine or masking [6]. Examining healthy volunteers, the same research group demonstrated that stimulation with an aversive sound leads to an increase in rCBF in the prefrontal cortex [26]. Kleinjung and colleagues [27] showed that tinnitus treatment with repetitive Transcranial Magnetic Stimulation (rTMS) applied on the temporal cortex can be enhanced by additionally stimulating the prefrontal cortex. Weisz et al. [7] reported that a reduction of alpha power and an enhancement of delta power in the prefrontal cortex correlates with tinnitus distress. The orbitofrontal cortex (OFC) in the tinnitus sample of the current study was characterized by enhanced influx from other regions and reduced output. The role of the orbitofrontal cortex in tinnitus has not been studied so far. Since other studies indicate that the OFC is part of the reward system [28-30], it is possible that it integrates the aversive information of the perceived tinnitus. This role of the OFC in this recording, however, remains speculative. The next cluster in this network was located in the posterior part of the brain including the occipital cortex, the parietal cortex, and the posterior cingulum. The outflow of this cluster was stronger in tinnitus subjects than in control participants. Another cluster approximately at the same position, but slightly more anterior was found to be significant for reduced inflow. Puzzling here is the outflow of the visual cortex. This might result from the coarse resolution with a voxel size of 2 cm that we used here. Since we used a standard-volume for all participants this adds imprecision to the mapping of the hubs. In a recently suggested model by Dehaene and colleagues, the parietal cortex and cingulate cortices have been associated with the global neuronal workspace and in the following we want to interpret the results in light of this framework.

This framework asserts the existence of workspace neurons that are distributed over the whole cortex, however, mainly in the parietal lobe, the frontal, the cingulate cortex and the sensory systems [9,10]. In order to form a conscious percept of a stimulus, two conditions are required: First, neuronal activity of the sensory cortex of the respective modality, and. second, an entry into the global neuronal workspace and thus long-range coupling between the widely distributed workspace neurons. According to this model, coupling within this fronto-parietal-cingulate network is needed for conscious perception (i.e. awareness of the stimulus). Activity of the sensory areas without this coupling would remain unconscious. In the present study, participants in the tinnitus group all reported a continuous perception of the tinnitus tone while the healthy participants in the control group did not report such a perception. Hence we would expect to find significant group differences in the coupling strength between global workspace neurons. The framework of the global workspace does not make any predictions on the frequency bands involved in this long-range cortical network. With the present study we found the inflow/outflow effects mainly in the gamma frequency range. This is in line with many other studies finding inter-regional coupling in the gamma frequency range and demonstrating its functional importance in the integration of information from widely distributed brain regions: Miltner et al. [31] revealed enhanced gamma band coupling during associative learning; Melloni et al. [32] used different masks to manipulate whether a test stimuli was visible or invisible to the participants. They detected significant differences of gamma phase locking between the *visible *and the *invisible *condition. In another study Supp and colleagues [21] visually presented familiar and unfamiliar objects and found different patterns of gamma long-range coupling between the two conditions.

Another assumption of the global workspace hypothesis is that top-down influence from the global workspace to the respective sensory region amplifies the neuronal activity there [9,10]. A top-down amplification of the neuronal activity in the auditory cortex in tinnitus has also been hypothesized earlier [8]. Indeed we found a significant correlation between the inflow to the temporal cortices and the subjective rating of the tinnitus distress: Tinnitus subjects with a stronger inflow to the temporal regions, report stronger distress. This explains why it has not been possible to reveal significant group differences between tinnitus and control participants regarding the temporal cortices. Since the degree of inflow to the temporal cortex of the tinnitus group was modulated by the tinnitus distress the variance in the tinnitus group was enhanced and the group difference did not reach significance. The outflow of the temporal cortices did not correlate with tinnitus distress. In a post-hoc analysis we were interested in the origin of this input to the temporal lobe, which we detected to stem largely from the prefrontal cortex, the parieto-occipital region, and regions adjacent to the left and right temporal cluster respectively. Thus, the top-down influence on the temporal cortex originates to a large extent from the prefrontal and the posterior clusters - clusters having been characterized before by an enhanced outflow in tinnitus. Because of methodological constraints with relatively large voxel sizes, a precise localization of the inflow cluster to specific anatomical structures within the temporal cortex is not possible. However, the localization of the maxima within the temporal clusters suggest that these clusters represents, at least partially, the auditory cortex. An involvement of other temporal structures (e.g. the hippocampus and parahippocampus) is also likely and cannot be excluded.

## Conclusion

In summary, we have found alterations in the long-range functional network in tinnitus subjects under rest which we assert to be related to the conscious perception of the distressing tinnitus tone. This network exerts top-down influence on the auditory cortices. The strength of this influence is associated with the subjective strength of the tinnitus distress. Repetitive Transcranial Magnetic Stimulation (rTMS) aims to reduce the hyperactivity in the auditory cortex which leads to a reduction of tinnitus loudness [33-37], however a complete relief of tinnitus is rare. On the other hand, cognitive therapies are also able to reduce tinnitus symptoms partially [38,39] and in light of the current study it can be argued that cognitive therapies alter the tinnitus-related global network and thus reduce the top-down influence of the global network on the temporal cortex. Overall we want to stress the importance of combining both branches of tinnitus therapy. Conceptually, a reduction of the hyperactivity in the auditory cortex cannot eliminate the tinnitus if the global network is still active and drives the tinnitus-related temporal activity. However, a reduction of the tinnitus-related global network activity cannot eliminate the tinnitus either if there is still an untreated abnormal pattern of spontaneous activity in the temporal cortex. It is hypothesized that sensory activity above a certain threshold can enter the global workspace in a bottom-up manner [9,10]. Thus, tinnitus therapy needs to *fight on two frontlines *at the same time: Reducing the hyperactivity in the auditory cortex on the one hand (e.g. via rTMS or Neurofeedback) and changing the global network on the other hand (e.g. via Tinnitus Retraining or meditation techniques).

## Methods

### Subjects

A total number of 47 participants took part in this study. They were all right-handed according to the Edinburgh Handedness Inventory (Oldfield, 1971) [40]. The study was approved by the institutional review board of the University of Konstanz. All participants were informed about the procedure and signed a written consent form prior to the measurement. The participants were recruited via the local newspaper and flyers posted at the University of Konstanz.

Twenty-three participants (mean age (± standard deviation): 43.9 years ± 18.4, five female) reported a perception of tinnitus while 24 healthy control participants (mean age: 45.4 years ± 14.1, 13 female) did not experience any tinnitus. All participants in the tinnitus group suffered from their tinnitus at least half a year (mean tinnitus duration: 4.25 years ± 3.5). Within this group, eight subjects experienced their tinnitus in the left ear, five individuals reported right-sided tinnitus, eight participants bilateral, and one person located his tinnitus in the middle of his head. Tinnitus Distress was assessed using the German version of the Tinnitus Questionnaire which is a widely used and neurophysiologically validated questionnaire for the subjective rating of tinnitus-related distress [24,41]. The total scale of this questionnaire ranges from 0 to 84 points with four distress categories: slight (0 to 30 points), moderate (31 to 46 points), severe (47 to 59 points), and very severe (60 to 80 points) distress. The average distress in our sample was 25.1 with a range from 3 to 59 points. More detailed information on the tinnitus sample is given in Table 1.

**Table 1:** Characteristics of the Tinnitus Group.

Patient	Age	Sex	Etiology	Tinnitus Distress	Tinnitus Duration	Tinnitus Side
1	47	m	Unknown	59	NA	NA
2	63	f	Unknown	59	NA	left
3	53	m	Stress	50	1	bilateral
4	58	m	Stress	22	11	bilateral
5	29	m	Unknown	NA	1	right
6	32	f	Unknown	5	2	right
7	22	f	Unknown	8	6	bilateral
8	23	m	Noise Trauma	3	3	bilateral
9	26	m	Lyme Disease	21	9	bilateral
10	25	f	Unknown	4	6	left
11	50	m	Noise Trauma	24	12	left
12	69	m	Trafic Accident	54	1.5	bilateral
13	50	m	Stress	16	3	left
14	43	m	Sudden hearing loss	25	1.5	left
15	47	m	Unknown	8	0.5	left
16	32	m	Unknown	9	NA	right
17	48	m	Rock Concert	17	2.5	bilateral
18	56	f	Stress	59	2.5	left
19	67	m	Stress	32	3	bilateral
20	48	m	Unknown	18	NA	right
21	42	m	Unknown	26	8	left
22	50	f	Unknown	13	NA	right
23	64	m	Stress	21	3	head

### Data acquisition

Neuromagnetic data were recorded with a 148-channel whole-head magnetometer system (MAGNES TM 2500 WH, 4D Neuroimaging, San Diego, USA) while the subjects lay in a comfortable supine position. The MEG-system was installed in a magnetically shielded and quiet room (Vakuumschmelze Hanau). The continuous data were recorded with a hard-wired high-pass filter of 0.1 Hz with a sampling rate of 678.17 Hz. In seven subjects we recorded with a sampling rate of 2,034.51 Hz. However, all data sets were down-sampled to 600 Hz prior to data analysis. The recording duration was set to five minutes and the subjects were asked to relax during this time, to stay awake with eyes open and not to engage in deliberate mental activity. Furthermore, they were instructed to fixate on a point at the ceiling of the measuring chamber and to avoid eye-movements as well as any body movements.

### Data analysis

Data preprocessing and most of the following steps of the data analysis were done using the fieldtrip toolbox (F. C. Donders Centre for Cognitive Neuroimaging: http://www.ru.nl/fcdonders/fieldtrip). First, all data sets were down-sampled to 600 Hz and cut into epochs of two seconds and those epochs containing blinks or muscle artifacts were excluded from further analysis based on visual inspection. Second, an independent component analysis (ICA) was calculated for each individual data set to identify components that reflect the heart-beat and these components were rejected from the data (using the logisitic infomax ICA algorithm implemented in eeglab: http://sccn.ucsd.edu/eeglab/). After artifact correction, 90 trials (i.e. 180 seconds in total) were selected randomly from the remaining trials and used for the following analyses. This selection was done to keep the number of trials constant across all subjects. The number of 90 trials reflects a trade-off between cleaning the data from noisy events as much as possible and still having enough data to calculate the autoregressive model.

#### Step 1: Source projection

To project the sensor data into source space, we used a linearly constrained minimum variance (LCMV; [42]) beamformer on each individual data set. The LCMV beamformer uses the covariance matrix of the single-trial signal data to construct a spatial filter that passes the signals for each time point to a predefined source while minimizing the contribution of other sources. The spatial filters were multiplied with the sensor time series, to derive the single-trial activities. The orientations were rotated such for each trial, that the first orientation accounted for a maximum of the signal. The orientations were then averaged across trials and applied to the single-trials. The subsequent analysis steps were then performed on the first orientation. A grid of 326 voxels (2 × 2 × 2 cm) that covers approximately the entire brain volume was used for the beamformer. We want to emphasize that, because of this relatively large voxel size, the allocation of the voxels to precise brain structures should be interpreted with caution.

#### Step 2: Partial directed coherence

For each subject, we computed partial-directed coherence (PDC) for the full set of voxels [19,20]. Partial- directed coherence is a measure of effective coupling that captures the direction of the information-transfer between the given voxels. Thus, with a set of N voxels, we get a total of NxN PDC-values for each subject that reflects for each pair of voxels the effective coupling in both directions. This approach is based on multivariate autoregressive (MVAR) modeling that integrates temporal and spatial information. Here, we model for each voxel the influence by all other voxels for a given time-range. The model order *p *defines this time range of the autoregressive process and describes how many time points - back in time - are used for the modeling the current value. In the univariate case this can be written as(1)

whereby *y(t) *denotes the predicted value at time-point *t*, *a(1), a(2),...a(p) *determine the regression coefficient and *x(t) *is called the *innovation process *which equals the difference between the actual value at time *t *and the estimation of *y(t) *based on the linear combination of the previous time points *y(t-1), y(t-2),... y(t-p) *[43]. In order to find the optimal model parameter *P *we calculated the Schwarz Bayesian Criterion (SBC) [44] for model orders from 2 - 20. On average over the whole sample, the minimum of the SBC function was located at *P *= 6 which was then taken as the model order for all subjects. For estimation of the autoregressive parameters we used the Vieira-Morf algorithm [45] implemented in the biosig toolbox (http://www.biosig.sf.net, version 2.12) which has been found to provide the most accurate estimates [43]. The matrix of autoregressive coefficients in the multivariate case can be written as(2)

where the coefficients *aij *represent the linear interaction between voxel *i *onto voxel *j *for a given time lag *k*.

Partial Directed Coherence is a statistical measure that is related to the concept of Granger Causality [46] and is able to detect asymmetric coupling between the compared voxels for a given frequency range. Here we investigated the frequency range of 2 to 100 Hz (steps of 2 Hz). In order to reveal the spectral properties, the autoregressive coefficients are transformed into the frequency domain by(3)

with  representing the matrix of the frequency-transformed autoregressive coefficents, *I *being the identity matrix and *f*_*s *_being the sampling frequency.  With denoting the *i, j*-th element of the relative coupling strength from voxel *j *to voxel *i *at a given frequency *f*, the directed information flow from *j *to *i *can be written by(4)

The superscript *H *denotes the Hermetian transpose which is found by taking the complex conjugate of each entry of the standard matrix transpose. Thus, the PDC value π ii *(f) *indicates how much the activity of voxel *i *depends on its own past at a given frequency. The value π ij *(f) *denotes how much the frequency-specific activity of voxel *j *depends on voxel *i*. The PDC estimators were calculated using functions implemented in the biosig toolbox (http://www.biosig.sf.net, version 2.12).

To the best of our knowledge, there is no established way of calculating the statistical significance of the PDC estimators. Thus, we used a permutation approach to estimate thresholds for significant coupling between pairs of voxels (couplings of one voxel with itself were excluded from the analysis). Therefore, the following steps 1) to 3) were repeated 1,000 times:

1) Shuffle the matrix A of the autoregressive coefficients pseudo-randomly. This was done the following way: The matrix A is a square matrix with 326 rows and 326 columns. Firstly, we generated a vector with random numbers between 1 and 326. Secondly, the columns were shuffled according to the random vector. Thirdly, the rows were shuffled according the same random vector. This shuffling procedure was repeated for all model orders.

2) Calculate the PDC estimators in the way that was described above.

3) Determine the 99%-percentile of the PDC estimator for each frequency and save it. The 99%-percentile was used instead of the maximum to reduce the influence of the self-reflective coefficients (voxel *i *with itself) which are much higher and were not part of this analysis anyway.

The maxima over the 1,000 permutations was used as a threshold of significance for each frequency bin. Thresholds were calculated for each participant individually.

#### Step 4: Hubmapping

Networks of any kind can be described by the distribution of their hubs. A node within a network that has a great number of connections with other nodes is called a *hub*. The degree of a node counts the number of connections and can be used as a measurement of the importance of a hub. In this analysis we weighted the degree of the hub by the strength of the couplings (i.e. the PDC estimator). Only significant couplings between pairs of voxels were used for the calculation of the hubs. Since Partial Directed Coherence allows an interpretation of the directionality of the coupling between two voxels we were able to differentiate between *Inflow *and *Outflow*. Thus, the degree of an *Inflow *is calculated by adding the significant PDC estimators of all voxels connection to this respective voxel. The hubdegrees for inflow and outflow were calculated for each frequency bin separately. They were mapped on the grid that was used for the beamformer for statistical analysis.

### Statistical analysis

#### Group comparison

The hubdegrees of the tinnitus and the control group were compared cluster-randomization approach [47,48]. This approach defines clusters of interest based on the actual distribution of the data and tests the statistical significance of these clusters using a *Monte-Carlo *randomization method with correction for multiple comparisons. Firstly, an independent samples t-test was calculated for each voxel between the tinnitus and the control group. This t-test was calculated for defining the clusters in the following step. Secondly, a cluster-finding algorithm was used to cluster the hubdegrees of neighboring voxels and neighboring frequency bins together that exhibit the same effect with a *P*-value < .05. The clustering was performed in space and frequency simultaneously. Clusters had to consist of at least two voxels. Thirdly, the t-statistic was calculated on a cluster-level by calculating the sum of t-values of the respective cluster. The maximum of this cluster-level statistics is taken to form the reference distribution over all randomizations. A total of 1,000 randomizations was done by shuffling the data of participants between groups. For each randomization the maximum cluster-t-value was saved to form a reference distribution of the cluster-t-values. The *P*-value of a cluster was estimated according to this reference distribution. The statistic for the inflow and the outflow was calculated separately.

#### Correlation analysis

The correlation between the subjective rating of the tinnitus distress and the inflow/outflow was calculated using the same cluster-randomization logic. The correlation coefficient was calculated for each voxel and frequency bin and the clusters were formed as described above. This time the permutation of the data was done within the tinnitus group by shuffling the hubdegree and the tinnitus distress rating of the respective patient.

## Abbreviations

EEG: Electroencephalography; ICA: Independent Component Analysis; lcmv: linearly constrained minimum variance; MEG: Magnetoencephalography; MVAR: Multivariate Autoregressive; OFC: Orbitofrontal Cortex; PDC: partial directed coherence; PET: Positron Emission Tomography; rCBF: regional cerebral blood flow; rTMS: repetitive Transcranial Magnetic Stimulation; SBC: Schwarz Bayesian Criterion.

## Authors' contributions

WS and NW participated in data collection, performed the analysis and drafted the manuscript. NM, TH, JK and IL participated in data collection, drafting the paper and discussion of the results.
